# Laparoscopic versus open approach in gastrectomy for advanced gastric cancer: a systematic review

**DOI:** 10.1186/s12957-020-01888-7

**Published:** 2020-06-13

**Authors:** Zhipeng Zhu, Lulu Li, Jiuhua Xu, Weipeng Ye, Junjie Zeng, Borong Chen, Zhengjie Huang

**Affiliations:** 1 Department of Gastrointestinal Surgery, Xiamen Cancer Center, The First Affiliated Hospital of Xiamen University, 55 Zhen Hai Road, Si Ming District, Xiamen, 361003 Fujian People’s Republic of China; 2 Department of Clinical Medicine, Fujian Medical University, Fuzhou, 350004 Fujian People’s Republic of China

**Keywords:** Systematic review, Meta-analysis, Gastrectomy, Laparoscopy, Advanced gastric cancer

## Abstract

**Background:**

Additional studies comparing laparoscopic gastrectomy (LG) versus open gastrectomy (OG) for advanced gastric cancer (AGC) have been published, and it is necessary to update the systematic review of this subject.

**Objective:**

We conducted the meta-analysis to find some proof for the use of LG in AGC and evaluate whether LG is an alternative treatment for AGC.

**Method:**

Randomized controlled trials (RCT) and high-quality retrospective studies (NRCT) compared LG and OG for AGC, which were published in English between January 2010 and May 2019, were search in PubMed, Embase, and Web of Knowledge by three authors independently and thoroughly. Some primary endpoints were compared between the two groups, including intraoperative time, intraoperative blood loss, harvested lymph nodes, first flatus, first oral intake, first out of bed, post-operative hospital stay, postoperative morbidity and mortality, rate of disease recurrence, and 5-year over survival (5-y OS). Besides, considering for this 10-year dramatical surgical material development between 2010 and 2019, we furtherly make the same analysis based on recent studies published between 2016 and 2019.

**Result:**

Thirty-six studies were enrolled in this systematic review and meta-analysis, including 5714 cases in LAG and 6094 cases in OG. LG showed longer intraoperative time, less intraoperative blood loss, and quicker recovery after operations. The number of harvested lymph nodes, hospital mortality, and tumor recurrence were similar. Postoperative morbidity and 5-y OS favored LG. Furthermore, the systemic analysis of recent studies published between 2016 and 2019 revealed similar result.

**Conclusion:**

A positive trend was indicated towards LG. LG can be performed as an alternative to OG for AGC.

## Introduction

Gastric cancer is one of the most common malignant diseases worldwide; the incidence and mortality for GC is still high [1]. Surgical resection with lymph node dissection is the most effective treatment for gastric cancer [2]. In the past, conventional open gastrectomy (OG) has been the mainstay of treatment for gastric cancer. Since laparoscopy-assisted gastrectomy was first described in 1994 [3], endoscopic and laparoscopic procedures for early gastric cancer have been increasingly used because of many advantages over OG, including less blood loss, fewer postoperative complications, faster bowel function recovery, shorter hospital stay, and an equivalent long-term outcomes [4–6]. The application of laparoscopic gastrectomy (LG) for advanced gastric cancer (AGC) was first reported [7]. Although several clinical trials have reported the effectiveness of LG [8, 9], considering of lacking of long-term oncological outcomes and the technical difficulties, there is no enough evidence to support LG for treating AGC; thus, the use of LG for AGC has been still controversial. Many previous meta-analyses have compared the short-term postoperative outcomes and long-term outcomes [10, 11], whereas they have analyzed the results without enough clinical randomized trials and/or with low-quality studies. Many high-quality RCTs related with LG treating AGC have been published recently, especially between 2018 and 2019. Therefore, we conducted this systematic review and meta-analysis to find some proofs for the use of LG in AGC.

## Materials and methods

### Search strategy

The comprehensive publications were identified by searching medical electronic databases PubMed, EMBASE, and Web of Science, which published from July 2010 to May 2019. The following MeSH terms and free-text terms were used: “laparoscopy-assisted gastrectomy”, “laparoscopic-assisted gastrectomy”, “laparoscopy surgery”, “laparoscopies”, “laparoscopic surgery”, “open gastrectomy”, “conventional gastrectomy”, “stomach neoplasms”, “gastric cancer”, “gastric neoplasm”, and “stomach cancer”; the Boolean operators “AND” and “OR” were used to combined these terms. The references of the relevant articles and previous meta-analysis studies were identified as additional articles. Title and abstracts of each identified article were screened, and the full text of the screened articles was assessed for eligibility. Three authors researched and reviewed independently and thoroughly through the above-mentioned search strategy; the search strategy was provided as supplementary file.

### Criteria of inclusion and exclusion

All included publications in this meta-analysis should meet following criteria:

1. Clinical studies containing RCTs and NRCTs (case-control study, and cohort study)
2. Clinical studies having compared LG versus OG for treatment of advanced gastric cancer
3. Detailed/available data of clinical studies have been reported, including short- or long-term data
4. Publication in English

All papers containing any of the following criteria were excluded:

1. Duplicate publication or the publication that did not provide sufficient data
2. No OG as a control group
3. Robot-assisted gastrectomy
4. Abstract only
5. Gastric surgery performed on benign lesions, non-primary gastric cancer, or recurrent gastric cancer
6. Patients in publication had non-curative factors such as distant metastasis of organs

### Data extraction

Clinical data was extracted independently and evaluated critically by two authors. Relevant data included characteristics of included study (author, year of publication, country of publication, study design, study period, male/female, age, tumor size, BMI, ASA (1:2:3)); summary of laparoscopic technique of included study (type of dissection, type of gastrectomy, retrieved LN, proximal margin, distal margin); systematic review of OS outcomes (follow-up (months), 5-y OS with relevant *P*); systematic review of recurrence pattern and sites; surgical outcomes including operative time, intraoperative blood loss, and harvested lymph nodes; recovery outcomes including time to first flatus, time to first oral intake, hospital stay, and mortality (defined as 30-day operative mortality); long-term outcomes including tumor recurrence and 5-year OS; and postoperative complications were classified as morbidity, overall complications, specific complications, and general complications. General complications included pneumonia, wound problems, postoperative ileus, and pancreatitis or pancreatic leakage; specific complications included intra-abdominal bleeding, anastomotic bleed, anastomotic stenosis, anastomotic leakage, duodenal stump leakage, abdominal infection, and lymphatic fistula.

### Quality assessment

To assess the quality of included studies, we used the Newcastle-Ottawa Quality Assessment Scale (NOS) for non-RCT [12]. NOS contains 3 categories including selection, comparability, and outcome, which were scaled by eight elements; high-quality elements are awarded by adding a star, no more than one stars could be added into the elements of selection and outcome, and no more than two stars could be added into the elements of comparability; then, studies were compared according to the number of stars, total score was 9 stars, 0–5 stars was considered as low-quality and 6–9 stars was considered as high quality. The risk of bias and quality of RCTs were determined by the Jadad scale (JCS) [13]. The high-quality trials should score ≥ 3 of a maximum possible score of 5.

### Statistical analysis

STATA 12.0 for Windows was performed for this study. Dichotomous data was calculated by relative risks (RR) with 95% confidence intervals, and continuous variables were calculated by weighted mean differences (WMD) with 95% (CI); 5-year OS was evaluated by pooled hazard ratios (HR) and their 95% CI. When the HR and 95% CI were not provided in the studies, some published formula were performed to calculate HR with 95% Cl [14]. A random effect model was used for studies with high heterogeneity, while a fixed-effect model was used for low heterogeneity. *χ*^2^ test was used to assess heterogeneity. Funnel plots and Egger’s linear regression test were used to assess the publication bias. *P* ≤ 0.05 was considered to indicate statistical significance.

## Result

### Results of the search and quality assessment

**Fig. 1:**
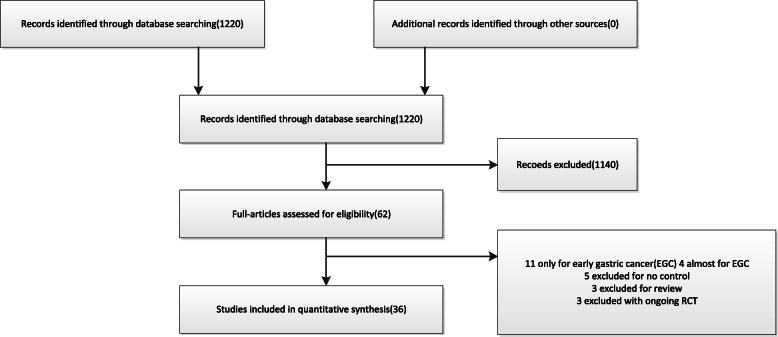
Flow diagram of the meta-analysis

The study selection process is summarized in the flowchart (Fig. 1). A total of 1220 publications were researched according to the search strategy, eighteen articles were excluded after duplication, and after titles, abstracts, and language were retrieved to assess further, 1140 publications were excluded. Of these studies, twenty-six studies were excluded because they included early gastric cancer only or almost early gastric cancer, were protocols of ongoing studies, were review, and were no control group. In the end, eighteen case-control studies, ten cohort studies, and eight RCT were enrolled in the studies [15–49]. In terms of non-RCT studies, four studies scored 6 (moderate-quality study) on the NOS, and twenty-four studies scored 8–9 (high-quality study) (Table 1). With regard to RCT, two studies scored 4 (high-quality study) on the JCS, and six studies scored 2 (moderate-quality) (Table 2).

**Table 1:** Assessment of quality of non-RCT studies (NOS)

References		Selection				Comparability		Outcome			
	Year	REC	SNEC	AE	DO	SC	AF	AO	FU	FUO	Score
Zhang et al. [40]	2017	*	*	*	*	*		*	*	*	8
Xu1 et al. [39]	2017	*	*	*	*	*	*	*	*	*	9
Lu et al. [37]	2016	*	*	*	*	*	*	*	*	*	9
Hao et al. [33]	2016	*	*	*	*	*		*	*	*	8
Zhang et al. [32]	2015	*	*	*	*	*		*	*	*	8
Wu et al. [31]	2015	*	*	*	*	*				*	6
Gordon et al. [25]	2013	*	*	*		*	*	*	*	*	8
Bo et al. [24]	2013	*	*	*	*	*	*	*	*	*	9
Chun et al. [20]	2012	*	*	*	*	*		*	*	*	8
Chen et al. [19]	2012	*	*	*	*	*	*	*	*	*	9
Zhao et al. [18]	2011	*	*	*	*	*		*	*	*	8
Shuang et al. [17]	2011	*	*	*	*	*		*	*	*	8
Shinohara et al. [27]	2013	*	*	*	*	*	*	*	*	*	9
Li3 et al. [42]	2018	*	*	*	*	*	*	*	*	*	9
Chan et al. [45]	2019	*	*	*	*	*	*	*	*	*	9
Fang et al. [28]	2014	*	*	*	*	*	*	*	*	*	9
Xu2 et al. [49]	2018	*	*	*	*	*	*	*	*	*	9
Huang et al. [14]	2010	*	*	*	*	*	*				6
Hamabe et al. [21]	2012	*	*	*	*	*	*	*	*	*	9
Scatizzi et al. [16]	2011	*	*	*	*	*		*	*	*	8
Inokuchi et al. [41]	2018	*	*	*	*	*	*	*	*	*	9
Li^1^ et al. [35]	2016	*	*	*	*	*	*				6
Zhang et al. [38]	2016	*	*	*	*	*	*	*	*	*	9
Kinoshita et al. [46]	2019	*	*	*	*	*	*	*	*	*	9
Kim et al. [22]	2012	*	*	*		*	*	*	*	*	8
Moisan et al. [23]	2012	*	*	*	*	*		*	*	*	8
Qiu et al. [29]	2014	*	*	*	*	*	*				6
Lin et al. [26]	2013	*	*	*	*	*	*	*	*	*	9

*REC* representativeness of the exposed cohort, *SNEC* selection of the non-exposed cohort, *AE* ascertainment of exposure, *DO* demonstration that outcome of interest was not present at start of study, *SC* study controls for age and sex, *AF* study controls for any additional factors, *AO* assessment of outcome, *FU* follow-up long enough for outcomes to occur, *FUO* adequacy of follow-up of cohorts

**Table 2:** Assessment of quality of RCTs (Jadad scale)

References	Year	Randomization	Blinding	Withdraw and dropout	Jadad’s score
Wang et al. [48]	2019	2	0	0	2
Lee et al. [47]	2019	2	0	0	2
Shi et al. [44]	2018	2	0	0	2
Park et al. [43]	2018	2	0	2	4
Li1 et al. [35]	2016	2	0	0	2
Hu et al. [34]	2016	2	0	2	4
Cai et al. [15]	2011	1	0	1	1
Cui et al. [30]	2015	2	0	0	2

Randomization: randomization was described with appropriate method—2 score, randomization was described without appropriate method—1 score, no randomization—0 score. Blinding: blinding was performed on all doctors and patients—2 score, blinding was partially performed on doctors and patients—1 score, no blinding—0 score. Withdraw and dropout: the reason of withdraw and dropout was described—1 score, the reason of withdraw and dropout was not described—0 score. Quality: high-quality trials should score ≥ 3, moderate-quality trials should score ≥ 2

### Characteristics of included study

According to the search strategy and criteria of inclusion and exclusion, a total of thirty-six studies published from 2010 to 2019 were eligible for the meta-analysis. A total of 11,808 cases (5714 cases in LAG and 6094 cases in OG) were involved in the study. Among the thirty-six studies, twenty-five studies originated from China, five originated from Japan, four originated from Korea, one originated from Italy, and one originated from Chile. Detailed information for characteristics of included study is shown in Table 3.

**Table 3:** Characteristics of included studies

References	Year	Country	Study design	Study period	Male/female		Age		Tumor size		BMI		ASA (1:2:3)
					L	O	L	O	L	O	L	O	
Huang et al. [14]	2010	Chi-	Cohort	2007–2008	40/26	39/30	55.80 ± 9.21	56.37 ± 10.63	-		–		–
Cai et al [15]	2011	Chi-	RCT	2008–2009	39/10	37/10	54.4 ± 10.6	52.6 ± 13.6	4.15 ± 2.03	4.32 ± 1.78	21.99 ± 3.29	22.87 ± 2.76	–
Scatizzi et al. [16]	2011	Italy	CCS	2006–2009	14/16	16/14	70 ± 12	69 ± 10.75	5.6 ± 3	7.2 ± 2.25	22 ± 1	24 ± 6.5	8/25/17
Shuang et al [17]	2011	Chi-	CCS	2005–2007	30/5	30/5	58 ± 10.5	59 ± 16	–		21 ± 3	23 ± 3	–
Zhao et al. [18]	2011	Chi-	CCS	2004–2009	248/98	221/92	51.43 ± 11.45	52.57 ± 12.37	4.4 ± 1.57	4.6 ± 1.72	–		–
Chun et al. [20]	2012	Korea	CCS	2004–2009	30/22	48/19	61.1 ± 12.6	60.8 ± 11.1	3.4 ± 1.6	4.0 ± 2.1	22.8 ± 2.8	22.9 ± 3.0	–
Hamabe et al. [21]	2012	Japan	Cohort	2000–2009	47/19	70/31	66.3	64.2	3.54	4.05	–		–
Kim et al. [22]	2012	Korea	CCS	2006–2007	53/35	50/38	56.0 ± 13.5	59.0 ± 13.1	3.8 ± 2.2	5.2 ± 2.7	22.9 ± 3.0	22.5 ± 3.0	–
Moisan et al. [23]	2012	Chile	Cohort	2003–2010	21/10	20/11	67 ± 13.5	67 ± 14	3.7 ± 1.975	4.5 ± 2.425	26 ± 2.75	25.5 ± 3.5	22/38/2
Bo et al. [24]	2013	Chi-	CCS	2004–2010	82/35	80/37	54.5 ± 10.6	52.6 ± 13.6	–		21.1 ± 3.0	21.7 ± 3.8	–
Gordon et al. [25]	2012	Japan	CCS	1999–2010	18/48	39/93	63.95 ± 12.11	66.96 ± 11.86	–		22.84 ± 3.45	22.06 ± 3.33	–
Lin et al. [26]	2013	Chi-	Cohort	2008–2010	12/71	12/71	61.6 ± 10.3	61.1 ± 10.5	4.6 ± 2.1	4.4 ± 2.2	22.3	21.5	–
Shinohara et al. [27]	2012	Japan	Cohort	1998–2008	129/57	85/38	61.4 ± 11.7	63.1 ± 9.9	–		21.5 ± 3.2	21.4 ± 3.3	173/127/9
Fang et al. [28]	2014	Chi-	CCS	2005–2009	78/9	78/9	57 ± 8.16	56 ± 7.66	4.4 ± 1.90	4.8 ± 1.50	23.3 ± 2.216	22.9 ± 2.216	69/81/24
Qiu et al. [29]	2014	Chi-	CCS	2012–2013	25/5	22/12	74.4 ± 3.1	75.6 ± 3.0	4.1 ± 1.9	4.9 ± 2.6	21.6 ± 2.7	21.6 ± 3.0	2/45/17
Cui et al. [30]	2015	Chi-	RCT	2010–2012	88/40	98/44	60.1 ± 12.6	57.5 ± 11.2	–		23.03 ± 3.61	23.66 ± 3.23	–
Wu et al. [31]	2015	Chi-	CCS	2010–2012	92/68	115/80	58.09 ± 10.87	58.61 ± 12.33	–		22.40 ± 3.85	22.20 ± 3.75	–
Zhang et al. [32]	2015	Chi-	CCS	2007–2014	57/29	61/25	62 ± 4.83	61 ± 4.00	–		–		107/49/16
Hao et al. [33]	2016	Chi-	CCS	2004–2011	437/191	414/165	54.57 ± 14.46	55.66 ± 12.59	–		–		610/520/77
Hu et al. [34]	2016	Chi-	RCT	2012–2014	380/129	346/174	56.5 ± 10.4	55.8 ± 11.1	4.0 ± 2.0	4.0 ± 2.1	22.7 ± 3.2	22.7 ± 3.2	–
Li1 et al. [35]	2016	Chi-	CCS	2012–2014	54/47	53/48	57.7 ± 10.5	59.5 ± 10.1	–		23.7 ± 71.1	23.7 ± 1.0	57/106/39
Li2 et al. [35]	2016	Chi-	RCT	2012–2014	13/7	27/3	53.5 ± 9.2	56.0 ± 9.2	3.7 ± 1.9	2.5 ± 1.3	22.4 ± 3.7	23.0 ± 3.1	1/38/5
Lu et al. [37]	2016	Chi-	CCS	2008–2015	39/22	37/24	59 ± 7.75	57 ± 6.75	–		19 ± 1.25	22 ± 2	88/26/8
Zhang et al. [38]	2016	Chi-	CCS	2009–2014	65/27	59/33	63 ± 5.66	65 ± 5.66	–		19 ± 1.66	22 ± 1.33	120/47/17
Xu1 et al. [39]	2017	Chi-	CCS	2007–2011	60/7	60/7	58.5 ± 10.5	57.7 ± 9.6	5.4 ± 2.3	5.8 ± 2.2	22.5 ± 3.2	22.6 ± 3.5	5/105/24
Zhang et al. [40]	2017	Chi-	Cohort	2006–2008	63/48	69/50	57.2 ± 11.2	58.7 ± 12.1	–		–		–
Inokuchi et al. [41]	2018	Japan	CCS	2001–2012	39/13	37/15	67 ± 10.25	68 ± 13	–		21.9 ± 3.65	22.1 ± 3.175	39/52/13
Li3 et al. [42]	2018	Chi-	Cohort	2007–2012	322/88	311/99	–		–		–		–
Park et al. [43]	2018	Korea	RCT	2010–2011	69/31	65/31	58.6	60.1	–		23.7 ± 3.0	23.3 ± 3.1	–
Shi et al. [44]	2018	Chi-	RCT	2010–2012	120/42	105/55	55.23 ± 11.01	55.02 ± 10.79	4.28 ± 1.94	4.40 ± 1.97	20.7 ± 3.086	20.2 ± 2.948	161/128/33
Xu2 et al. [49]	2018	Chi-	Cohort	2005–2012	342/88	595/173	55.6 ± 10.4	56.8 ± 10.6	4.7 ± 1.8	4.9 ± 1.8	22.4 ± 3.0	22.2 ± 3.2	165/873/157
Chan et al. [45]	2019	Hong Kong	Cohort	2009–2017	35/19	110/57	70 ± 12.5	66 ± 9.5	–		–		13/136/71
Chen et al. [19]	2012	Chi-	CCS	2008–2010	49/175	23/89	61.6 ± 10.6	60.8 ± 10.2	4.7 ± 2.0	4.4 ± 2.0	22.3	22	180/143/13
Kinoshita et al. [46]	2019	Japan	Cohort	2008–2014	214/91	217/88	67.1	67.7	–		21.5 ± 3.1	21.6 ± 3.4	195/359/56
Lee et al. [47]	2019	Korea	RCT	2011–2015	333/127	321/137	59.9 ± 10.8	59.5 ± 11.6	4.5 ± 2.4	4.5 ± 2.2	23.5 ± 2.9	23.7 ± 2.3	445/431/42
Wang et al. [48]	2019	Chi-	RCT	2014–2017	144/78	133/87	59.4 ± 12.6	60.6 ± 10.2	3.6 ± 1.8	3.9 ± 1.2	23.1 ± 3.1	23.5 ± 3.5	179/255/8

*RCT* randomized controlled trial, *CCS* case-controlled study, *L* laparoscopic gastrectomy, *O* open gastrectomy, *TG* total gastrectomy, *DG* distal gastrectomy, *PG* proximal gastrectomy, *–* not available

### Summary of laparoscopic technique

All the included studies have reported the laparoscopic technique. Thirty-four studies have demonstrated the details on the level of lymphadenectomy, D2 lymphadenectomy was performed in 29 studies, D1, D1+, D2, and D2 + lymphadenectomy were used in three studies, D1 + α/β and D2 were used in one study, and D0, D1, D1 + α/β, D2, and D2+ were performed in one study. All the studies have reported the type of gastrectomy; compared with proximal and subtotal gastrectomy, distal and total gastrectomy were frequently used for advanced gastric cancer. Twelve studies reported the resection margin in both LADG and ODG groups, only one article showed there was significant difference in proximal margin between the two groups [21], and all the rest indicated no significant difference between the two groups for proximal margin and distal margins. Thirty-five studies have shown detailed data of retrieved LN between the two groups, thirty-two studies indicated no significant difference, while three studies showed *P* < 0.05. Detailed information for characteristics of included studies is shown in Table 4. Relevant pathological characteristics of included studies are shown in Table 5.

**Table 4:** Summary of laparoscopic technique

Reference	Type of dissection	Type of gastrectomy	Retrieved LN			Proximal margin			Distal margin		
			L	O	P	L	O	P	L	O	P
Huang et al. [14]	D2	DG	25.81 ± 12.53	27.47 ± 0.28	0.401	-			-		
Zhao et al. [18]	D1,D1 + α, β, D2, D2+	DG	33.2 ± 12.5	32.8 ± 15.6	0.715	6.25 ± 2.04	6.29 ± 2.11	0.805	5.68 ± 1.71	5.62 ± 1.59	0.642
Shuang et al. [17]	D2	DG	35 (7–63)	38 (6–66)	NS	–			–		
Scatizzi et al. [16]	D2	DG	37 (8–89)	31 (16–60)	0.174	–			–		
Cai et al. [15]	D2	PG DG TG	22.98 ± 2.704	22.8 ± 2.428	0.839	–			–		
Chun et al. [20]	D2	DG	39.1 ± 15.2	39.3 ± 11.2	0.971	5.0 ± 2.9	6.0 ± 3.6	0.014	6.0 ± 3.4	5.4 ± 3.1	0.372
Chen et al. [19]	D2	DG TG	30.6 ± 10.1	30.3 ± 8.6	0.786	–			–		
Hamabe et al. [21]	D2	DG TG	63.7 ± 26.4	44.0 ± 18.9	< 0.0001	–			–		
Kim et al. [22]	D2	TG SG TG	38.3 ± 14.3	41.8 ± 15.3	0.1187	4.4 ± 3.0	4.5 ± 3.1	0.8695	7.3 ± 4.8	7.2 ± 5.4	0.9363
Moisan et al. [23]	D1 + α, D1 + β, D2	TG SG	35 (9–68)	39 (12–109)	0.805	–			5.5 (0.3–13.5)	5.5 (0.3–13.5)	0.982
Gordon et al. [25]	D0, D1, D1 + α, D1 + β, D2, D2+	DG	35.92 ± 12.60	36.5 ± 14.48	0.739	–			–		
Bo et al. [24]	–	TG	35.2 ± 11.7	37.4 ± 13.2	0.132	3.5 ± 1.2	3.2 ± 0.9	0.517	–		
Shinohara et al. [27]	D2	DG PG TG	45.3 ± 16.9	43.8 ± 17.2	0.446	–			–		
Lin et al. [26]	D2	TG DG	30.2 ± 10.1	28.0 ± 8.1	0.103	–			–		
Fang et al. [28]	D2	TG DG	32 (8–65)	36 (12–72)	NS	–			–		
Qiu et al. [29]	D2	DG TG	30.2 ± 12.0	28.1 ± 11.8	0.484	–			–		
Zhang et al. [32]	D2	DG TG	20 (16–23)	21 (17–23)	0.58	–			–		
Wu et al. [31]	D2	DG PG TG	19.84 ± 4.7	18.04 ± 4.14	NS	6.33 ± 1.91	6.44 ± 2.04	0.621	5.73 ± 1.47	5.92 ± 1.11	0.149
Cui et al. [30]	D2	PG DG TG	29.3 ± 11.8	30.1 ± 11.4	0.574	–			–		
Lu et al. [37]	D2	TG	18 (17-23)	19 (16-24)	0.548	–			–		
Hao et al. [33]	D1,D1+,D2, D2+	DG PG TG	30.4 ± 13.4	28.1 ± 17.1	0.43	6.15 ± 1.63	6.09 ± 1.09	0.54	5.46 ± 1.74	5.40 ± 1.95	0.57
Li et al [35]	D2	DG	33.7 ± 7.1	33.1 ± 7.6	0.358	–			–		
Zhang et al. [38]	D2	DG TG	17 (16–21)	18 (17–25)	0.258	–			–		
Li et al. [35]	D2	DG	24.7 ± 8.3	24.6 ± 10.0	0.967	5.8 (5.0, 6.8)	5.0 (4.0, 7.5)	0.476	3.0 (2.0, 3.8)	3.0 (2.0, 4.0)	0.634
Hu et al. [34]	D2	DG	36.1 ± 16.7	36.9 ± 16.1	0.738	4.8 ± 2.3	5.2 ± 2.5	0.436	4.1 ± 2.1	4.3 ± 2.5	0.239
Zhang et al. [40]	–	DG PG TG	37 ± 14	35 ± 11	0.05	–			–		
Xu et al. [39]	D2	DG TG	6.0 ± 6.9	6.4 ± 7.2	0.353	4.8 ± 2.5	4.6 ± 2.4	0.354	6.6 ± 4.2	7.6 ± 4.7	0.105
Li et al. [42]	D2	DG TG	–			–			–		
Xu et al. [49]	D2	DG TG	21.6 ± 8.6	22.4 ± 10.3	0.136	5.0 ± 2.8	5.4 ± 2.9	0.066	7.2 ± 4.3	6.9 ± 4.6	0.292
Inokuchi et al. [41]	D2	DG TG	39 (14–72)	38 (14–89)	0.69	–			–		
Shi et al. [44]	D2	PG DG TG	31.59 ± 5.87	32.18 ± 6.07	0.377	–			–		
Park et al. [43]	D2	DG	37.0 ± 13.4	39.7 ± 13.3	0.168	–			–		
Chan et al. [45]	D2	TG	37 (7–77)	26 (3–95)	< 0.001	–			–		
Kinoshita et al. [46]	D1, D1+,D2, D2+	DG PG TG	43 (32–56)	34 (24–44)	< 0.001	–			–		
Wang et al. [48]	D2	DG	29.5 ± 10.4	31.4 ± 12.3	0.083	5.0 ± 2.2	5.3 ± 2.5	NS	3.8 ± 2.4	3.9 ± 2.7	NS
Lee et al. [47]	D2	DG	46.6 ± 17.7	46.9 ± 15.9	0.741	4.6 ± 3.0	5.0 ± 3.1	0.053	4.8 ± 3.2	4.8 ± 3.1	0.87

*NS* not significant, – not reported, *LN* lymph nodes, *L* laparoscopic, *O* open

**Table 5:** Pathological characteristics of included studies

	LG			OG			LG				OG				LG					OG				
	Upper	Middle	Lower	Upper	Middle	Lower	T1	T2	T3	T4	T1	T2	T3	T4	N0	N1	N2	N3a	N3b	N0	N1	N2	N3a	N3b
Zhang et al. [32]																								
Zhang et al. [40]	30	18	63	35	24	60																		
Zhao et al. [18]																								
Zhang et al. [38]	10	23	59	12	26	54																		
Xu et al. [49]	185	190	391	97	114	195		148	371	249		97	195	98	178	159	176	191	64	117	99	92	96	26
Xu et al. [39]															13	11	21	15	7	15	11	18	16	17
Wu et al. [31]																								
Wang et al. [48]	2	29	181	2	35	173	58	45	65	54	52	35	71	62	100	43	30	49		93	43	39	45	
Shuang et al. [17]		21	14		22	13		15	20			13	22		23	5	7			19	11	5		
Shinohara et al. [27]	42	94	46	26	63	34	25	96	65		17	58	48		73	65	45	3		44	49	28	1	
Shi et al. [44]	35	46	81	35	39	86		30	132			35	125		47	61	35	19		33	66	40	21	
Scatizzi et al. [16]		10	15		9	16	0	9	20	1	0	9	20	1	5	11	14			6	13	11		
Qiu et al. [29]	3	11	16	6	7	21																		
Park et al. [43]																								
Moisan et al. [23]	12	7	12	13	10	8	15	4	5	7	15	4	5	7	22	2	4	3		23	2	4	3	
Lu et al. [37]																								
Lin et al. [26]	24	17	42	29	11	43		30	53			30	53		30	17	17	19		29	20	15	19	
Li et al. [36]																								
Li et al. [36]	62	10	29	62	10	29																		
Li et al. [42]								108	252	99		152	473	231	138	88	100	104	29	190	170	183	219	94
Lee et al. [47]							140	104	137	132	127	114	137	120	226	96	90	64	9	222	103	73	74	26
Kinoshita et al. [46]	266	395	436	195	190	266		148	531	479		39	253	374										
Kim et al. [22]	14	18	56	18	16	53		44	26	18		41	23	24	50	16	17	5		46	10	21	11	
Inokuchi et al. [41]																								
Huang et al. [14]																								
Hu et al [34]															214	87	88	129		216	79	98	126	
Hao et al. [33]	108	162	358	101	165	313		218	410			196	383		188	134	306			144	135	300		
Hamabe et a. [21]	17	28	21	24	40	37	21	45			18	83												
Gordon et al. [25]	18	48		88	44																			
Fang et al. [28]								23	42	22	25	39	23		38	21	17	11		32	23	22	10	
Cui et al. [30]																								
Chun et al. [20]	39	13		55	12										33	9	9	1		35	12	12	8	
Chen et al. [19]								81	143			50	62		81	42	47	54		41	25	25	21	
Chan et al. [45]	23	20	11	48	47	72	23	28	3			59	85	23	24	30				74	93			
Cai et al. [15]								24	25			21	26											
Bo et al. [24]	64	23	30	65	21	31	50	67				47	70		29	42	46			26	47	44		

*LG* laparoscopic gastrectomy, *OG* open gastrectomy

### Operative results

**Fig. 2:**
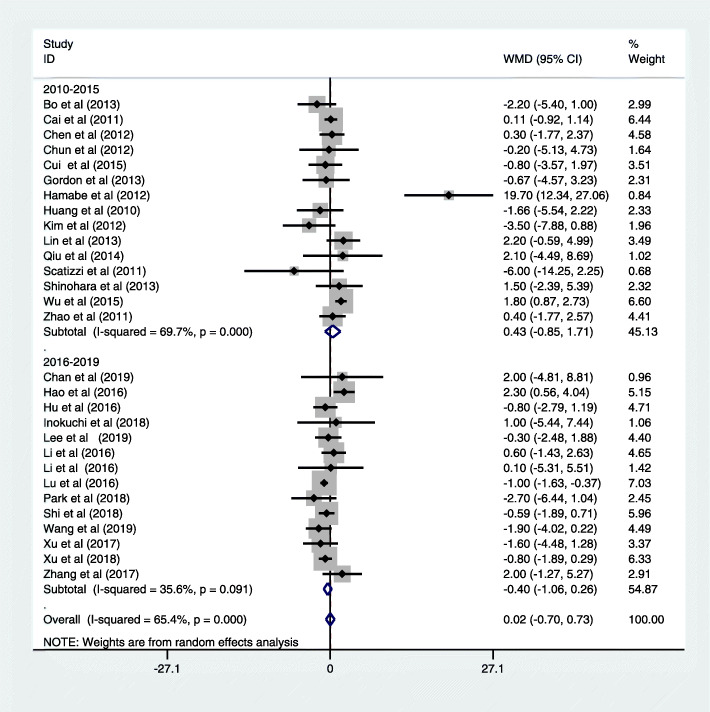
Forest plot of harvested lymph nodes

Table 6 showed the surgical outcomes of both types of surgery. Twenty-five studies reported the data of intraoperative blood loss [15, 16, 19, 21, 22, 25–28, 30–36, 38, 40, 42, 43, 45–47, 49, 50], twenty studies demonstrated that LG was significantly associated with less blood loss in the operation [15, 19, 20, 22, 25–28, 30–35, 38, 42, 45, 46, 49, 50], and only one study demonstrated the opposite result [47]. Twenty-nine studies report the data of operative time [15–17, 19–22, 25–28, 30–36, 38, 40, 42–47, 49–51], the duration of LG was significantly longer than that of OG in twenty-three studies [15, 17, 21, 22, 25, 26, 28, 30–32, 34–38, 40, 42, 44, 45, 47, 49–51], but only one study demonstrated the opposite result [46]. Twenty-nine studies report the data of retrieved lymph [15–17, 19–22, 25–28, 30–36, 38, 40, 42–47, 49–51], twenty-seven have reported the number of retrieved lymph nodes in LG was similar to that in OG [15–17, 19–21, 25–28, 30, 31, 33–36, 38, 40, 42–47, 49–51], and two studies showed that the number of harvested lymph nodes was significantly higher for LG than OG [22, 32]. Our analysis showed that LG could produce satisfactory result, which indicated that lymph node dissection could be carried out with laparoscopic surgery (WMD = 0.02, 95% CI = − 0.70, 0.73; *P* > 0.05; Fig. 2).

**Table 6:** Surgical outcomes of LG and OG

Reference	Blood loss (ml)		Operating time (min)		Harvested lymph node	
	LAG	OG	LAG	OG	LAG	OG
2010–2015						
Huang et al. [14]	131.91^a^	342.3	266.05^a^	223.78	25.81	27.47
Cai et al. [15]	293.67	344.47	270.51	187.66	22.98	22.87
Scatizzi et al. [16]	NA	NA	240^a^	180	31	37
Zhao et al. [18]	128^a^	301	211	204	33.2	32.8
Chen et al. [19]	82.7^a^	201.7	207.2	213	30.6	30.3
Chun et al. [20]	NA	NA	207.7^a^	159.9	39.1	39.3
Hamabe et al. [21]	158.3^a^	356.3	283.1^a^	225.9	63.7^a^	44
Kim et al. [22]	NA	NA	228.3^a^	183.6	38.3	41.8
Bo et al. [24]	196.9^a^	358.2	292.8^a^	242.1	35.2	37.4
Gordon et al. [25]	107^a^	495	291^a^	235	35.92	36.59
Lin et al. [26]	78.4^a^	200.4	212.7	226.4	30.2	28
Shinohara et al. [27]	154.3^a^	388.7	369.7^a^	263.6	45.3	43.8
Qiu et al. [29]	120^a^	227.3	259.5^a^	236.09	30.2	28.1
Cui et al. [30]	99^a^	125	258^a^	194	29.3	30.1
Wu et al. [31]	169.46^a^	193. 86	228.43^a^	207.59	19.84^a^	18.04
2016–2019						
Hao et al. [33]	154.5^a^	311.2	257.8^a^	231	30.4	28.1
Hu et al. [34]	105.5^a^	117.3	217.3^a^	186	36.1	36.9
Li1 et al. [35]	131.9	129.5	297.4^a^	198.1	33.7	33.1
Li2 et al. [35]	94	97.9	214.2^a^	200.3	24.7	24.6
Lu et al. [37]	250^a^	330	240^a^	190	18	19
Xu1 et al. [39]	322	274	326^a^	203	24	25.6
Zhang et al. [40]	143^a^	223	189	201	37	35
Inokuchi et al. [41]	115^a^	420	316^a^	242	39	38
Park et al. [43]	NA	NA	257.4^a^	183	37	39.7
Shi et al. [44]	129^a^	215.8	238.1^a^	207.3	31.59	32.18
Xu2 et al. [49]	273.7^a^	233.6	283.8^a^	191.5	21.6	22.4
Chan et al. [45]	150^a^	275	321^a^	365	39.5	37.5
Lee et al. [47]	138.3^a^	222	225.7^a^	162.3	46.6	46.9
Wang et al. [48]	91.4^a^	117.5	242.5^a^	209.9	29.5	31.4

*LG* laparoscopy gastrectomy, *OG* open gastrectomy

^a^*P* < 0.05

Considering for this 10-year dramatical surgical material development between 2010 and 2019, we make a subgroup analysis based on published year (2010–2015 and 2016–2019). Thirteen recent studies published between 2016 and 2019 reported intraoperative blood loss [34–38, 40–42, 45–47, 49, 50], ten studies indicated significantly less intraoperative blood loss in LG [34, 35, 38, 41, 42, 45–47, 49, 50], and no study reported opposite result. Recent fourteen studies reported operative time [34–38, 40–42, 44–47, 49, 50], and thirteen studies indicated the duration of LG was significantly longer than that of OG [34–38, 40, 42, 44–47, 49, 50]. All recent fourteen studies revealed that LG was similar to OG in retrieved lymph nodes [34–38, 40–42, 44–47, 49, 50], and subgroup analysis focused on 2016–2019 demonstrated no significant difference in lymph node dissection (WMD = − 0.40, 95% CI = − 1.06, 0.26; *P* > 0.05; Fig. 2). Furthermore, we make a subgroup analysis based on clinical study type; lymph node dissection showed no significant difference between the two groups in the RCT group (WMD = − 0.69, 95% CI = − 1.45, 0.07; *P* > 0.05; Figure S1) and non-RCT (WMD = 0.39, 95% CI = − 0.55, 1.32; *P* < 0.05; Figure S1). Besides, we make a subgroup analysis based on the type of gastrectomy; similar lymph node dissection was found between the two groups in distal gastrectomy (DG) (WMD = − 0.63, 95% CI = − 1.46, 0.21; *P* > 0.05; Figure S2) and total gastrectomy (TG) (WMD = − 1.22, 95% CI = − 4.70, 2.26; *P* > 0.05; Figure S2).

### Postoperative recovery

In terms of postoperative recovery, LG was also superior to OG (Table 7). Twenty-two studies reported a significantly shorter hospital stay after LG than OG [15, 17, 19, 20, 25–28, 30, 32–36, 38, 40, 42, 45, 46, 49–51]; four studies showed hospital stay in LG was similar to that in OG [16, 21, 44, 47]. Nineteen demonstrated that first flatus returned earlier after LG with statistical significance [15, 17, 19, 20, 26, 27, 30, 32–36, 38, 40, 43, 45, 47, 49, 51]; six studies showed that first flatus in LG was similar to that in OG [16, 21, 25, 42, 44, 50]. Seven studies indicated that first out of bed returned earlier after LG with statistical significance [19, 25, 28, 30, 34, 35, 45]; four studies showed that first out of bed in LG was similar to OG [16, 17, 20, 49]. Fourteen individual studies reported a significantly earlier first oral intake after LG than OG [17, 19, 20, 25, 27, 28, 30, 32, 34–36, 42, 45, 49]; four studies showed that first oral intake in LG was similar to that in OG [15, 16, 43, 50].

**Table 7:** Recovery outcomes of LG and OG

Reference	Hospital stay (days)		First flatus (days)		First out of bed (days)		First oral intake	
	LAG	OG	LAG	OG	LAG	OG	LAG	OG
2010-2015								
Huang et al. [14]	9.2^a^	11.35	3.18^a^	4.5	NA	NA	6.53	7.64
Cai et al. [15]	11.6327	11.4255	3.89	4.2128	4.7755	4.8936	6.8571	6.4681
Scatizzi et al. [16]	7^a^	9	2^a^	3	1	1	3^a^	4
Zhao et al. [18]	7.9^a^	10.7	3^a^	3.9	3^a^	4.3	3.5^a^	4.5
Chen et al. [19]	13.3^a^	17.4	2.6^a^	3.2	2.7	2.9	4.7^a^	5.1
Chun et al. [20]	7	7	3.1	3.1	NA	NA	NA	NA
Kim et al. [22]	7^a^	10.4	3.2^a^	3.7	NA	NA	NA	NA
Bo et al. [24]	7.4^a^	10.7	3.4	3.9	3.1^a^	5.3	4.5^a^	5.3
Gordon et al. [25]	8.4^a^	18.1	2.7^a^	3.8	NA	NA	NA	NA
Lin et al. [26]	14.2^a^	17.2	2.9^a^	4	NA	NA	4.1^a^	5.5
Shinohara et al. [27]	16.3^a^	24.3	NA	NA	2^a^	3.2	3.4^a^	5.7
Qiu et al. [29]	13^a^	16.9	2.9^a^	4.6	1.2^a^	4.1	4.5^a^	5.5
Wu et al. [31]	9.44^a^	11.07	3.72^a^	4.41	NA	NA	5.66^a^	7.09
2016–1019								
Hao et al. [33]	7.6^a^	10.7	3.3^a^	3.9	3.1^a^	4.5	3.7^a^	4.5
Hu et al. [34]	10.8^a^	11.3	1.4^a^	3.6	2.3^a^	2.4	5.5^a^	6
Li1 et al. [35]	10.5^a^	11.9	2.8^a^	3.6	NA	NA	3.8^a^	4.6
Li2 et al. [35]	10.875	10.625	3.2^a^	3.9	NA	NA	6.357	6.25
Lu et al. [37]	8^a^	10	2^a^	4	NA	NA	NA	NA
Xu1 et al. [39]	10.7	10.2	4.4^a^	4.8	NA	NA	NA	NA
Zhang et al. [40]	8.6^a^	13.2	2.3^a^	3.5	NA	NA	NA	NA
Inokuchi et al. [41]	9^a^	12	3	4	NA	NA	2^a^	4
Park et al. [43]	9.8	9.1	2.6	2.5	NA	NA	NA	NA
Shi et al. [44]	7.51^a^	10.49	3.14^a^	3.96	3.15^a^	4.37	3.57^a^	4.41
Xu2 et al. [49]	8.2^a^	8.7	4^a^	4.4	NA	NA	NA	NA
Chan et al. [45]	9^a^	11	NA	NA	NA	NA	NA	NA
Lee et al. [47]	8.1^a^	9.1	3.5	3.7	NA	NA	3.7	3.8
Wang et al. [48]	9.9^a^	10.9	2.8^a^	3.1	1.2	1.4	7^a^	7.9

*LG* laparoscopy gastrectomy, *OG* open gastrectomy

^a^*P* < 0.05

In terms of the studies published between 2016 and 2019, eleven studies indicated significantly shorter hospital stay in LG than OG [34–38, 40–42, 44–47, 49, 50]; three studies reported the similar result between LG and OG [37, 40, 44]. Compared with OG, the first flatus returned earlier with statistical significance in recent ten studies for LG [34–38, 40, 41, 45, 47, 49], and three studies indicated no significant difference between LG and OG [42, 44, 50]. Three significant studies showed that first out of bed returned earlier in LG compared with OG [34, 35, 45], and one study revealed no statistical difference between the two groups [49]. For the first oral intake, six studies demonstrated a significant result for LG [34–36, 42, 45, 49], and two studies indicated LG was similar to OG [37, 50].

### Postoperative morbidity and mortality

**Fig. 3:**
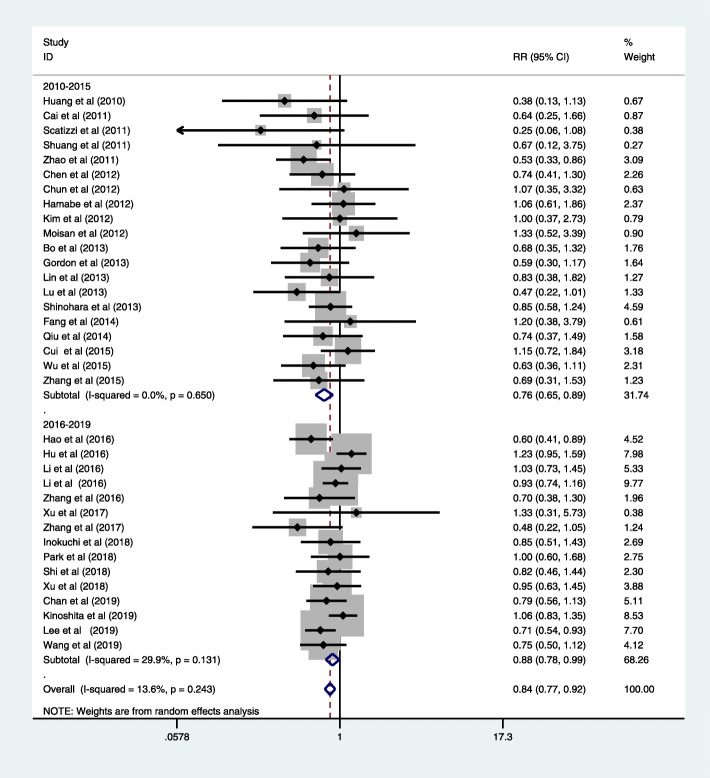
Forest plot of morbidity

The data from thirty-five studies indicated the rate of overall postoperative complications was lower in LG (RR = 0.84, 95% CI = 0.78, 0.92, *P* < 0.05) [15–42, 44–49]; the result was associated with low-grade heterogeneity between studies (Fig. 3, Table 8). In terms of the studies published between 2016 and 2019, fifteen studies present data in favor of LG (RR = 0.88, 95% CI = 0.78, 0.99, *P* < 0.05) [34–37, 39–42, 44–47, 49, 50]. We furtherly performed a subgroup based on clinical study type, the result favored LG in the non-RCT group (RR = 0.82, 95% CI = 0.74, 0.91, *P* < 0.05; Figure S3), and RCT group indicated that LG has similar postoperative complications to OG (RR = 0.92, 95% CI = 0.77, 1.13, *P* < 0.05; Figure S3). Moreover, we make a subgroup analysis based on the type of gastrectomy; the result of LG was not inferior to OG in TG (RR = 0.77, 95% CI = 0.56, 1.05, *P* > 0.05; Figure S4) and DG (RR = 0.82, 95% CI = 0.68, 1.00, *P* < 0.05; Figure S4).

**Table 8:** Analysis of postoperative morbidity

	Sample size							Heterogeneity test	
Measure outcomes	No. of studies	LG	OG	OR, WMD, or HR	Lower 95% CI	Upper 95% CI	*P*	*I*^2^ (%)	*P*
Over morbidity	35	79	1031	0.83	0.77	0.91	> 0.05	14.50	0.228
Specific complications									
Intra-abdominal bleeding	12	23	28	0.86	0.5	1.47	> 0.05	0	0.838
Anastomotic bleed	12	14	13	1.14	0.59	2.22	> 0.05	0	0.919
Anastomotic stenosis	11	19	24	0.75	0.42	1.31	> 0.05	0	0.747
Anastomotic leakage	24	75	97	0.85	0.63	1.15	> 0.05	0	0.819
Duodenal stump leakage	13	27	29	0.93	0.56	1.52	> 0.05	0	0.845
Abdominal infection	16	56	69	0.78	0.55	1.1	> 0.05	0	0.996
Lymphatic fistula	8	16	17	0.86	0.45	1.64	> 0.05	0	0.896
General complications									
Pneumonia	20	106	132	0.84	0.65	1.08	> 0.05	0	0.88
Wound problems	22	79	143	0.53	0.41	0.7	< 0.0001	3.50	0.413
Postoperative ileus	15	35	57	0.64	0.43	0.96	< 0.05	0	0.89
Pancreatitis or pancreatic leakage	14	64	42	1.42	0.98	2.05	> 0.05	0	0.935

*LG* laparoscopic gastrectomy, *OG* open gastrectomy

**Fig. 4:**
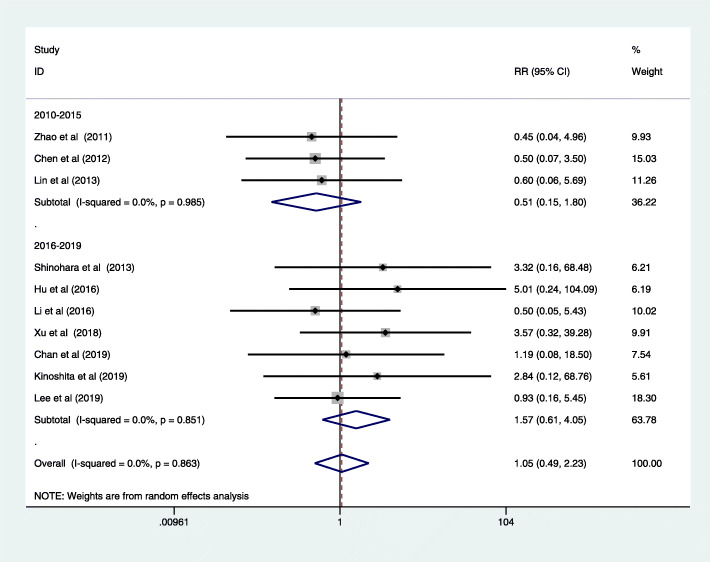
Forest plot of 30-day mortality

**Fig. 5:**
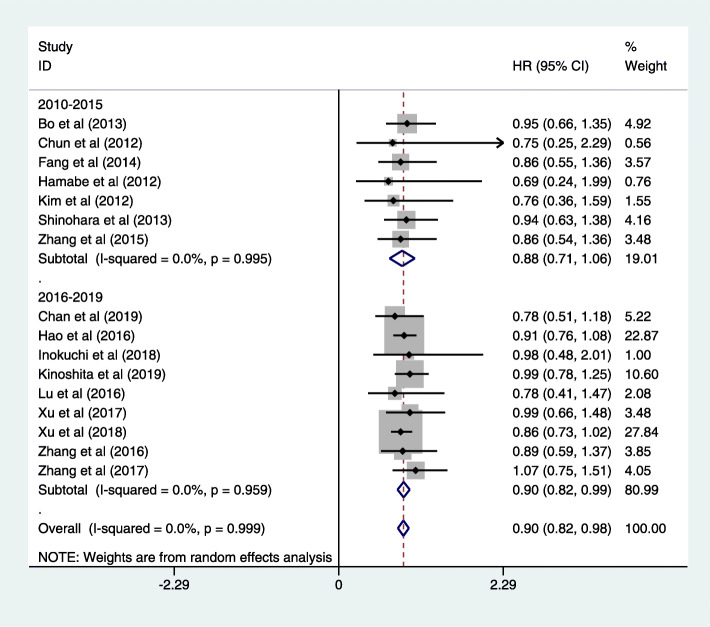
Forest plot of 5-year overall survival

The subgroup analysis of postoperative complications showed that significantly lower incidence rate of wound problems (RR = 0.53, 95% CI = 0.41, 0.70; *P* < 0.05) and postoperative ileus (RR = 0.64, 95% CI = 0.43, 0.96; *P* < 0.05) in LG group, and there was no significant difference in other surgery complications, including pneumonia, intra-abdominal bleeding, anastomotic bleed, anastomotic stenosis, anastomotic leakage, duodenal stump leakage, abdominal infection, lymphatic fistula, pancreatitis, or pancreatic leakage (Table 8). Ten articles reported the post-operative mortality (RR = 1.27, 95% CI = 0.57, 2.82; *P* > 0.05) [19, 20, 27, 28, 35, 36, 40, 46–48], with no significant difference and heterogeneity among these included articles (*I*^2^ = 0; *P* = 0.819). There were also no significant difference in post-operative mortality between analyzed groups for recent studies (RR = 1.57, 95% CI = 0.61, 4.05; *P* > 0.05, Fig. 4) [28, 35, 36, 46, 47, 50]. The subgroup analysis based on clinical study type indicated no significant difference in the non-RCT group (RR = 1.05, 95% CI = 0.41, 2.67, *P* > 0.05; Fig. 5) and RCT group (RR = 1.05, 95% CI = 0.29, 3.80, *P* > 0.05; Figure S5). The subgroup analysis based on operative procedure also indicated no significant difference in DG (RR = 0.83, 95% CI = 0.19, 3.64, *P* > 0.05; Figure S6) and TG (RR = 1.19, 95% CI = 0.08, 18.50, *P* > 0.05; Figure S6).

### Long-term postoperative outcomes

Follow-up ranged widely from 1 month to 149.4 months. Sixteen trials contain the data of 5-year overall survival [21–23, 25, 28, 29, 33, 34, 38–42, 46, 47]. The results were in favor of the LG group (HR = 0.91, 95% CI = 0.83, 0.98; *P* < 0.05), with moderate grade between the two groups (*I*^2^ = 65.4%, *P* = 0.999, Fig. 5). Thirty-five studies reported no significant difference in the over survival rate. The systematic review of long-term outcomes is summarized in Table 9. We furtherly make a subgroup analysis based on published years. As for the studies published in 2010–2015, there was no significant difference in terms of 5-year overall survival (HR = 0.88, 95% CI = 0.71, 1.06; *P* < 0.05) [21–23, 25, 28, 29, 33]. However, studies published in 2016–2020 revealed LG was associated with better result (HR = 0.90, 95% CI = 0.82, 0.99; *P* < 0.05) [34, 38–42, 46, 47]. Subgroup analysis based on operative procedure also indicated no significant difference between LG and OG in DG (RR = 0.86, 95% CI = 0.62, 1.10, *P* > 0.05; Figure S7). There was only one study in the TG group, and it also did not report a significant difference (RR = 0.75, 95% CI = 0.18, 1.77; Figure S7).

**Table 9:** Systematic review of OS outcomes

References	Group	Follow-up (mo.)	OS	*P*
Zhao et al. [18]	L	37 (6–72)	1 y, 87.2%; 3 y, 57.2%; 5 y, 50.3%	NS
	O		1 y, 87.1%; 3 y, 54.1%; 5 y 49.2%	
Shuang et al. [17]	L	36.5 (23–50)	50 mo., 64%	NS
	O	38.5 (27–50)	50 mo., 60%	
Scatizzi et al. [16]	L	18 (2–37)	42 mo., 70.91%	0.449
	O	18 (7–42)	42 mo., 56.77%	
Cai et al. [15]	L	22.1354 (4–36)	40 mo., 67.1%	–
	O		40 mo., 53.8%	
Chun et al. [20]	L	60.4 (7.0–91.7)	5 y, 91.3%	0.613
	O	53.2 (1.0–82.2)	5 y, 88.6%	
Chen et al. [19]	L	19 (1–48)	1 y, 91.5%	0.297
	O		1 y, 89.8%	
Hamabe et al. [21]	L	30.4 (1–60.9)	5 y, 94.4%	0.4877
	O	53.5 (1.3–111.3)	5 y, 78.5%	
Kim et al. [22]	L	53.7 (8.3–138.1)	5 y, 85.9 %	0.463
	O	58.1 (0.3–106.2)	5 y, 83.1%	
Moisan et al. [23]	L	28 mo.	3 y, 82.3%	0.557
	O	40 mo.	3 y, 86.9	
Gordon et al. [25]	L	49.2 (4–146)	5 y, 79.2%	NS
	O		5 y, 77.2%	
Bo et al. [24]	L	61.2 mo. (6–84 mo.)	5 y, 49.3%	0.756
	O		5 y, 46.5%	
Shinohara et al. [27]	L	48.8 (25–58.5)	5 y, 68.1 %	0.968
	O		5 y, 63.7 %	
Lin et al. [26]	L	23.0(12-50 )	1 y, 88.0%	NS
	O		1 y, 85.5%	
Fang et al. [28]	L	44 (1-82)	5 y, 59%	5 .525
	O		5 y, 54%	
Zhang et al. [32]	L	38	5 y, 59%	0.523
	O	40	5 y, 56%	
Hao et al. [33]	L	53.5	5 y, 57.65%	0.22
	O		5 y, 53.69%	
Li1 et al. [35]	L	–	–	–
	O	–	–	
Zhang et al. [38]	L	38	5 y, 57%	0.606
	O	40	5 y, 50%	
Zhang et al. [40]	L	37 (3–60)	1 y, 89.2 %; 3 y, 72.1%; 5 y, 45.7%	NS
	O		1 y, 87.4%; 3 y, 68.1%; 5 y, 42.3%	
Xu et al. [39]	L	22(3-100)	5 y, 31.3%	0.949
	O		5 y, 29.9%	
Li3 et al. [42]	L	69(3–120)	5 y, 52.0%	0.805
	O		5 y, 53.4%	
Xu et al. [49]	L	58 (0–129)	5 y, 51.2%	0.081
	O	49.5 (0–104.5)	5 y, 46.7%	
Inokuchi et al. [41]	L	62.2 (2.8–149.4)	5 y, 70%	0.96
	O	62.2 (4.4–130.4)	5 y, 73%	
Park et al. [43]	L	38.2	–	–
	O			
Chan et al. [45]	L	25	60 mo., 47%	0.233
	O	35	60 mo., 39%	
Kinoshita et al. [46]	L	3.4 y (1.3–5.0)	5 y, 54.2%	–
	O	3.5 y (1.7–5.0)	5 y, 53.0%	

*OS* over survival, *DFS* disease-free survival, *NS* not significant, – not report, *y* year, *mo*. month

**Fig. 6:**
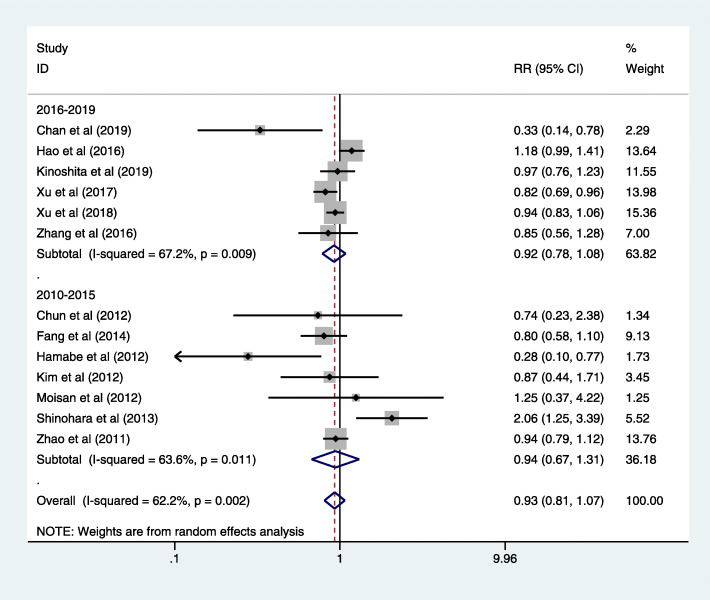
Forest plot tumor recurrence

No statistical difference was found between the LG and OG groups in tumor recurrence (RR = 0.93, 95% CI = 0.81, 1.07; *P* > 0.05) [19, 21–24, 28, 29, 34, 39, 40, 46, 47], with moderate-grade heterogeneity (*I*^2^ = 62.2%; *P* = 0.002) (Fig. 6). Besides, we analyzed the data from studies published in 2016–2019; there showed no significant difference between LG and OG (RR = 0.94, 95% CI = 0.67, 1.31; *P* > 0.05) [19, 21–24, 28, 29]. Furthermore, subgroup analysis based on operative procedure also indicated no significant difference between LG and OG in DG (RR = 0.94, 95% CI = 0.79, 1.10, *P* > 0.05; Figure S8). There was only one study in the TG group, and it showed lower incidence rate of tumor recurrence in LG (RR = 0.33, 95% CI = 0.14, 0.78; Figure S8). In the studies reporting the site of recurrence, local recurrence was the most frequent recurrence site among these common sites; relevant data related with specific recurrent sites are shown in Table 10.

**Table 10:** Systematic review of recurrence pattern and sites

Reference	Group	Size	Total	local	LN	Live	Peritoneum	Port site/wound	Bone	Hematogenous	Lung	Anastomotic stoma	Gut	Brain	Pancreas	Multiple pattern	Other
Zhao et al. [18]	L	346	147	–	–	–	–		–	–						–	
	O	313	141	–	–	–	–		–	–						–	
Chun et al. [20]	L	52	6	–	3	2	–	–	1	–						–	
	O	67	11	1	5	1	3	–	1	–						–	
Hamabe et al. [21]	L	66	5	0	0	0	4	–	0	–	1					–	
	O	101	21	1	7	9	3	–	1	–	0					–	
Kim et al. [22]	L	88	28	1	4	6	3	–	3	4		2				5	
	O	88	23	4	3	4	1	–	0	7		2				2	
Moisan et al. [23]	L	31	5	–	–	–	–	–	–	–						5	
	O	31	4	–	–	–	–	–	–	–						4	
Bo et al. [24]	L	117	23	–	–	4	15	–	1	–						3	
	O	117	26	–	–	2	20	–	–	–						4	
Shinohara et al. [27]	L	186	85	15	15	–	29	–	–	23						–	
	O	123	54	11	11	–	17	–	–	15						–	
Fang et al. [28]	L	87	36	–	–	–	–	–	–	–						–	
	O	87	45	–	–	–	–	–	–	–						–	
Lu et al. [37]	L	61	19	7	3	–	6	0	–	3						–	
	O	61	20	8	3	–	7	0	–	2						–	
Hao et al. [33]	L	628	196	12	20	–	45	–	–	30						–	
	O	579	158	9	11	–	42	–	–	23						–	
Zhang et al. [38]	L	92	28	2	3	5	9	4	1	–	1			3			
	O	92	32	2	2	5	10	7	1	–	1			4			
Xu et al. [39]	L	67	49	–	–	–	–	–	–	–						–	
	O	67	60	–	–	–	–	–	–	–						–	
Li3 et al. [42]	L	410	115	44	10	–	51	–	–	25							17
	O	410	101	51	13	–	49	–	–	20							23
Xu et al. [49]	L	430	203	97	–	42	6	2	12	–	16	10	9	2	1		5
	O	768	387	175	–	77	12	0	24	–	26	33	16	7	5		12
Chan et al. [45]	L	54	5	–	–	–	–	–	–	–						–	
	O	167	47	–	–	–	–	–	–	–						–	
Kinoshita et al. [46]	L	261	100	3	21	20	42	–	4	–	4						6
	O	258	121	4	32	22	45	–	3	–	5						10

*NS* not significant, – not reported, *LN* lymph nodes, *L* laparoscopic, *O* open

## Discussion

Between 2010 and 2019, there are many high-quality RCTs that have been published. However, many previous meta-analyses have included low-quality studies or a limited number of studies [52, 53]. Thus, we conducted the meta-analysis to evaluate whether LG is an alternative treatment for AGC. Some primary endpoints were compared between LG and OG, including intraoperative time, intraoperative blood loss, harvested lymph nodes, proximal and distal resection margin distance, time to first flatus, time to first oral intake, post-operative hospital stay, complication and mortality, rate of disease recurrence, and 5-year over survival.

The LG consumed significantly more time than OG, although we could get a wider operation field by applying LG. However, the operative process is more complicated and less flexible than OG; some reasons include the narrow operating field, restriction in the number of trocar [54], lacking of tactile sensation [55], insufficient training [56], the time for setting up the equipment, and the complexity of performing the esophagojejunostomy [57], while LG combined with advanced techniques for systemic lymphadenectomy may be the main reason, which needs experienced surgeons. In terms of AGC, compared with gastrectomy alone, gastrectomy combined with systemic lymphadenectomy is more complicated. Meanwhile, compared with other laparoscopic surgery including laparoscopic colectomy and cholecystectomy, LG with lymphadenectomy is also more difficult because it is necessary to identified many important vessels and clear lymph node. Recently, some studies have indicated that the operative time could significantly reduce and reach a plateau after about 40 cases, and the operative time of LG is no longer than OG for extensive technical expertise [51, 58, 59]; meantime, some high-quality studies reported postoperative morbidity has no significant difference between LG and OG, with LG leading to faster postoperative recovery [60]. With the development and improvement of laparoscopic techniques, the operative time will reduce and become shorter.

In spite of the operative time is longer, blood loss is significantly less in LG. For LG, by using the laparoscopic device such as ligatures and ultrasonic scalpel, we could get enlarged surgical vision to detect large and small vessels and expose vessel adequately, which contribute to small blood loss. The small amount of blood loss may contribute to a decreased blood transfusions, which could reduce the postoperative complication such as lung injury, volume overload, and pneumonia. Thus, small amount of blood loss has an impact on postoperative recovery and recurrence [61].

The postoperative complication is usually used to evaluate the surgical safety. The meta-analysis demonstrated that the overall postoperative complication rate of LG was significantly lower than OG; meantime, the wound problem and postoperative ileus were significantly less common than OG, which is in consistent with some previous meta-analysis [62, 63]. For LG, the smaller surgical surface wound and less manual handling may account for less wound problem, and LG could reduce the intervention to microenvironment of abdominal cavity and intestinal serous membrane, which may decrease the rate of postoperative ileus. The rate of postoperative pneumonia was lower than OG with no significant difference. In terms of OG, some disadvantages may make it difficult to cough, which lead to respiratory complications such as pneumonia, including tension sutures, serious pain, and abdominal bandages, while patients in LG were related with less blood loss, less blood transfusion during surgery [64, 65], and less wound pain after surgery [66]. For other postoperative complications including pancreatitis/pancreatic leakage, intra-abdominal bleeding, anastomotic bleeding, anastomotic stenosis, anastomotic leakage, duodenal stump leakage, abdominal infection, and lymphatic fistula, there were no significant differences.

With regard to the time to first flatus, the time to first oral intake, and post-operative hospital stay, the results were favoring for LG. LG is thought to be a less invasive procedure with smaller surgical incision and minimal gastrointestinal interference, so that the postoperative pain is less during recovery with a reduced inflammatory response and better glucose tolerance [67, 68], which has a direct impact on a quick recovery of bowel function, and a quick recovery represents earlier oral intake, earlier discharge, and shorter hospital stay. In other words, it is quicker to return to normal condition in LADG than ODG. The cost of laparoscopic surgery is higher for LG compared with OG, because of the costs of the disposable instruments, while Miura et al. [69] indicated that LADG was less expensive than ODG because hospital stay is shorter and additional costs can be offset by the lower charges for ward, meals, and nursing care.

In terms of oncological safety, in most previous studies, the number of HLNs is widely considered as the index of “quality” [70–75]; adequate LN dissection could reduce the possibility of recurrence and metastasis. The efficiency for lymphadenectomy is still the main concern; the efficiency represents surgical removal of fifteen lymph nodes is the minimum standard. In most previous studies and our present studies, the mean number of harvested lymph nodes was more than fifteen for LG. However, whether laparoscopy could reach the same result as open surgery is still controversial; a previous study showed that experienced surgeons could realize the radicality in lymphadenectomy if the operative time is not limited [76]. In the present meta-analysis, we discovered that the harvested lymph nodes have no significant difference, which indicated LG could retrieve as many LNs as did OG through the improvement in laparoscopy facilities and sufficient training. D1+α or β dissection is now adequate for selected patients with early gastric cancer. With regard to advanced gastric cancer, whether D2 dissection is superior to D1 dissection remains controversial [77, 78]. D2 dissection could realize more radical lymphadenectomy than D1 dissection, whereas D2 dissection could increase the postoperative mobility and mortality because of the invasiveness. In East Asia, maybe the incidence is high so that Asian surgeons are familiar with gastric cancer and have a better understanding of surgical technique; D2 lymphadenectomy is generally accepted as the standard to treat AGC. The Japanese Gastric Cancer Association has presented D2 lymphadenectomy as the standard treatment of local AGC [79]. However, some western studies have reported no significant long-term advantage with higher operative morbidity and mortality rates after D2 lymphadenectomy [80–82], and western surgeons would like to perform D1 lymphadenectomy; many recent report have indicated that patients who underwent laparoscopic gastrectomy combined with systemic lymphadenectomy could get a good survival result by extensively trained western surgeons [83, 84]. Therefore, insufficient training of the laparoscopic gastrectomy combined with systemic lymphadenectomy may be the main reason for western surgeons. D2 dissection is an appropriate treatment for patients with advanced disease. Proximal esophageal and distal duodenal margins is also used to assess oncological adequacy, and proximal and distal margin distance could reflect the radicality of surgery, which is related with recurrence and OS and DFS in GC and other cancer [85]. Meantime, surgical margin is considered as an independent prognostic factor for GC. Our analyses also showed that there was no significant difference between the two groups; it indicated that LG is oncologically acceptable for proximal or distal located tumors.

Long-term outcome is the most useful endpoint to evaluate the oncological safety and effectiveness of surgery. Tumor recurrence and 5-year OS are usually used to evaluated the long-term outcome. Many studies have showed tumor recurrence was similar between the LADG and ODG [86, 87]; previous clinical studies and meta-analysis have revealed that there was no significant difference in the 5-year OS between LADG and ODG [89]. In our meta-analysis, we make subgroup analysis based on published year; there demonstrated no statistically significant difference between the two groups; however, the results of 5-year OS favor LG with significant difference between 2016 and 2020, and the tumor recurrence showed no significant difference between the two groups. In other words, at least, LG is not inferior to ODG in terms of oncologic outcomes, which is acceptable for treatment of AGC.

Some limitations exist that should not be neglected for this meta-analysis. Many studies related with the theme are non-randomized retrospective trials; therefore, we have analyzed both the RCTs and NRCTs to avoid lack of samples. Several drawbacks of methodology may lead to heterogeneity, although the study has no observed obvious heterogeneity. In many included studies, the patients with multiple tumor stages were incorporated into one group and included in a single survival curve; it will have a significant effect on the quality and results of the meta-analysis. More ongoing RCTs should be performed to resolve the problem in the future.

## Conclusion

In conclusion, we make a systematic review of thirty-six studies to release LG could be considered to be expanded in treating AGC. Gastric cancer is difficult to diagnose early with a poor prognosis, and patients often do not realize until cancer progresses to middle and advanced stages. Our study has presented the safety and curability of LG, which indicated an encouraging result for LG to be widely accepted in the future. More ongoing RCTs comparing the LAG with OG should be recommended.

## Supplementary information

**Additional file 1: Figure S1.** Subgroup analysis based on clinical study type for lymph node dissection.

**Additional file 2: Figure S2.** Subgroup analysis based on the type of gastrectomy for lymph node dissection.

**Additional file 3: Figure S3.** Subgroup based on clinical study type for postoperative complications.

**Additional file 4: Figure S4.** Subgroup analysis based on the type of gastrectomy for postoperative complications.

**Additional file 5: Figure S5.** Subgroup analysis based on clinical study type for post-operative mortality.

**Additional file 6: Figure S6.** Subgroup analysis based on operative procedure for post-operative mortality.

**Additional file 7: Figure S7.** Subgroup analysis based on operative procedure for 5-year overall survival.

**Additional file 8: Figure S8.** Subgroup analysis based on operative procedure for tumor recurrence.

## Data Availability

The datasets used and/or analyzed during the current study are available from the corresponding author upon reasonable request.

## Abbreviations

LG: Laparoscopic gastrectomy
OG: Open gastrectomy
AGC: Advanced gastric cancer
5-y OS: 5-Year over survival
RCT: Randomized controlled trials
NRCT: Retrospective studies
NOS: Newcastle-Ottawa Quality Assessment Scale
JCS: Jadad scale
RR: Relative risks
WMD: Weighted mean differences
HR: Hazard ratios
DG: Distal gastrectomy
TG: Total gastrectomy
