# Supplementary figures and images for: Laparoscopic versus open approach in gastrectomy for advanced gastric cancer: a systematic review
Source: World J Surg Oncol. 2020 Jun 13;18:126. doi: 10.1186/s12957-020-01888-7 (PMC7293787; doi:10.1186/s12957-020-01888-7)

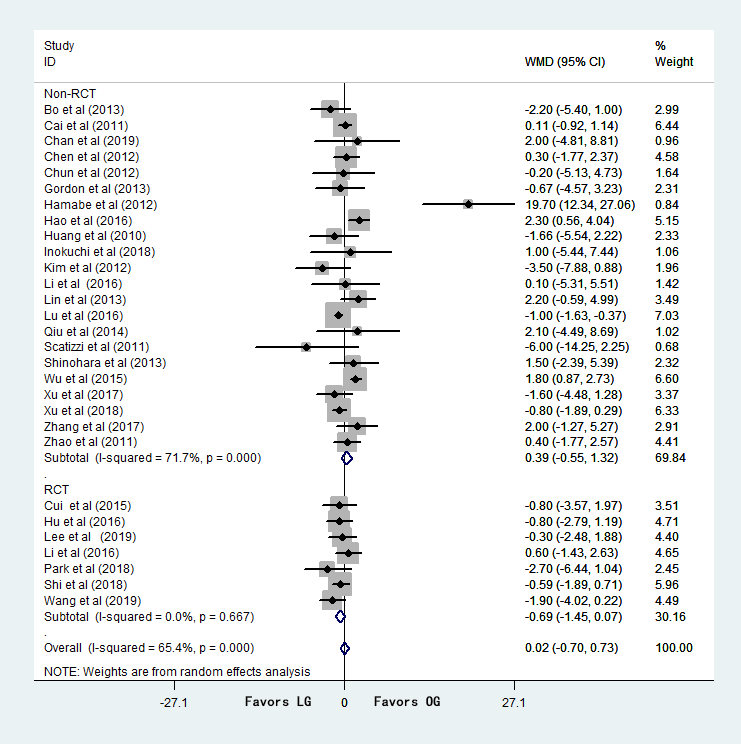

Supplement: Supplementary file 1 — Additional file 1: Figure S1. Subgroup analysis based on clinical study type for lymph node dissection. [file 12957_2020_1888_MOESM1_ESM.tif]

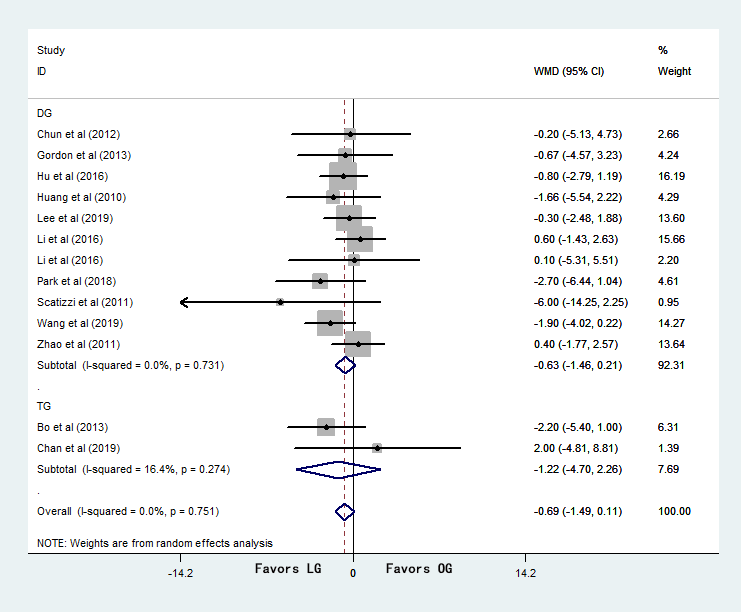

Supplement: Supplementary file 2 — Additional file 2: Figure S2. Subgroup analysis based on the type of gastrectomy for lymph node dissection. [file 12957_2020_1888_MOESM2_ESM.tif]

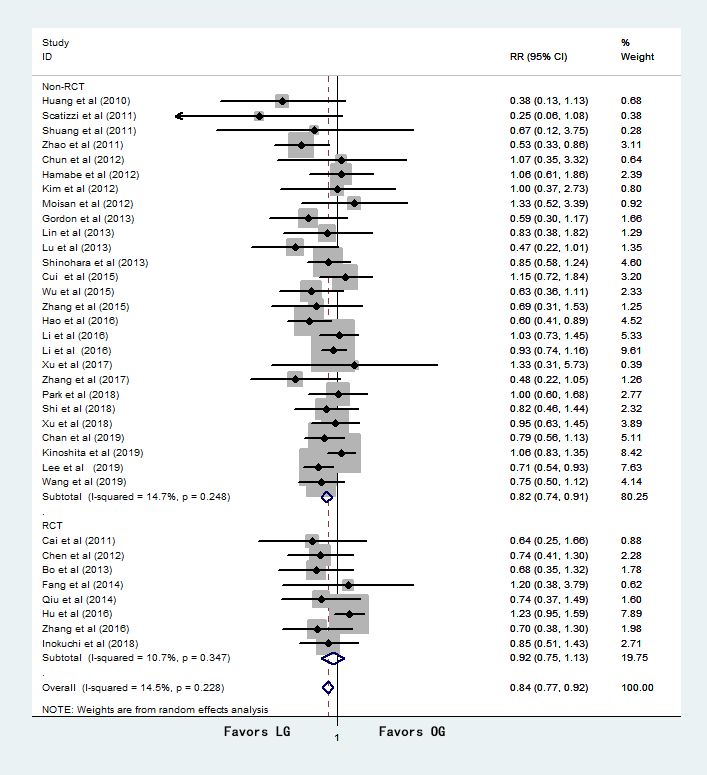

Supplement: Supplementary file 3 — Additional file 3: Figure S3. Subgroup based on clinical study type for postoperative complications. [file 12957_2020_1888_MOESM3_ESM.tif]

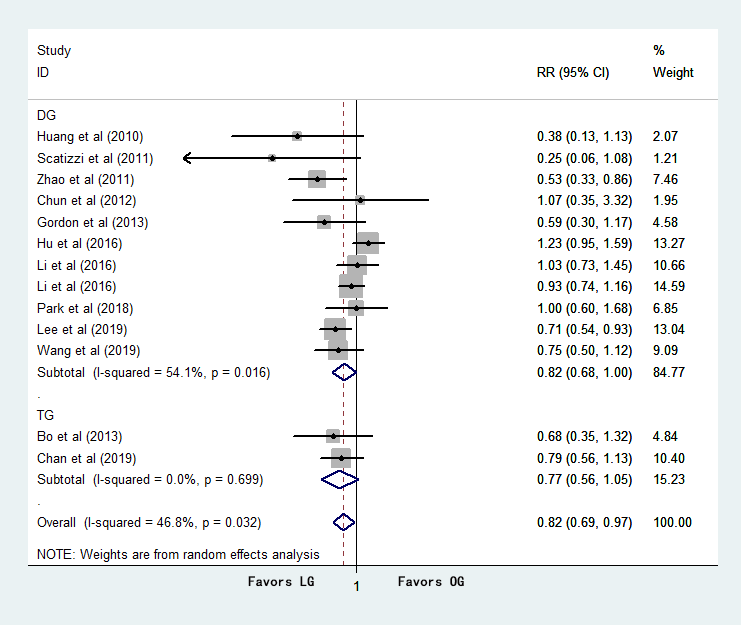

Supplement: Supplementary file 4 — Additional file 4: Figure S4. Subgroup analysis based on the type of gastrectomy for postoperative complications. [file 12957_2020_1888_MOESM4_ESM.tif]

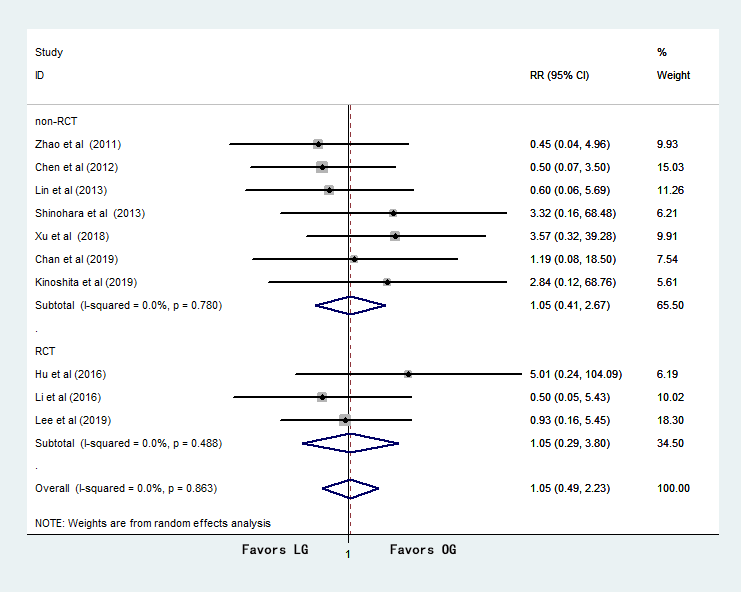

Supplement: Supplementary file 5 — Additional file 5: Figure S5. Subgroup analysis based on clinical study type for post-operative mortality. [file 12957_2020_1888_MOESM5_ESM.tif]

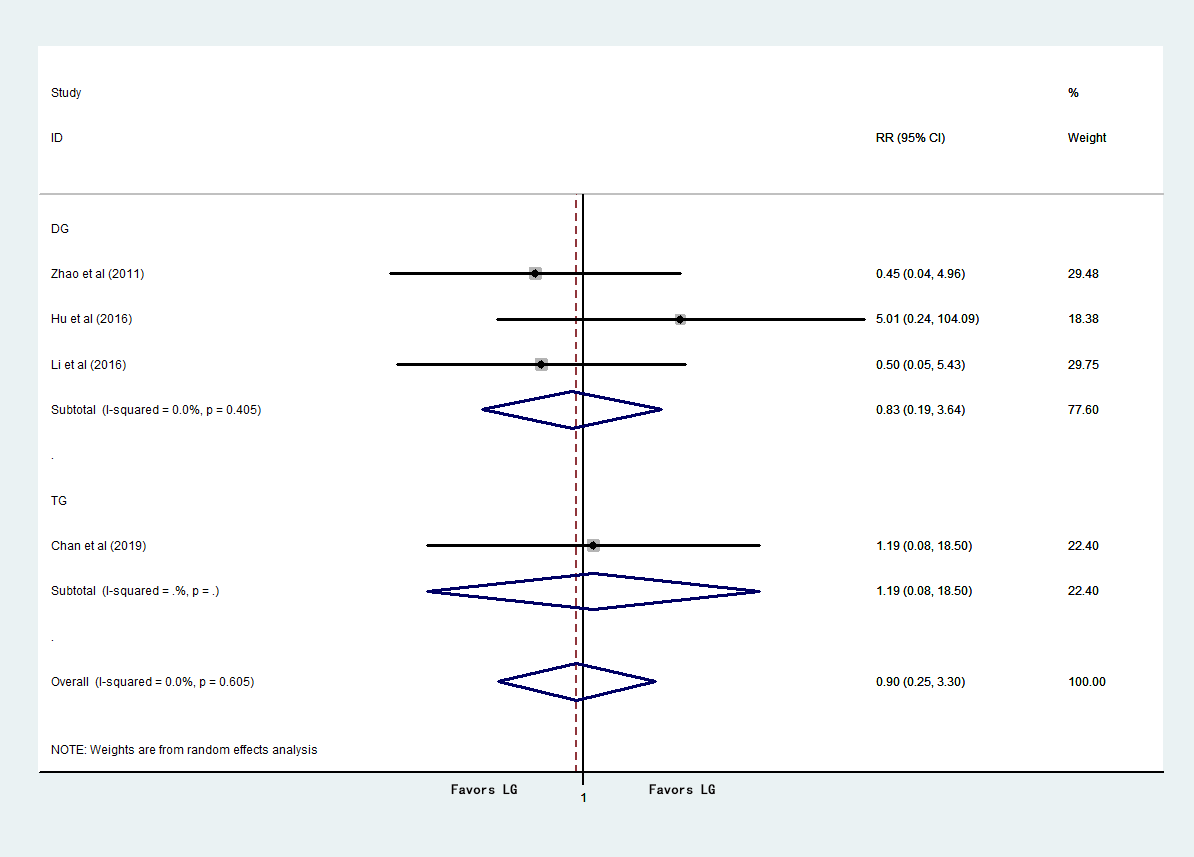

Supplement: Supplementary file 6 — Additional file 6: Figure S6. Subgroup analysis based on operative procedure for post-operative mortality. [file 12957_2020_1888_MOESM6_ESM.tif]

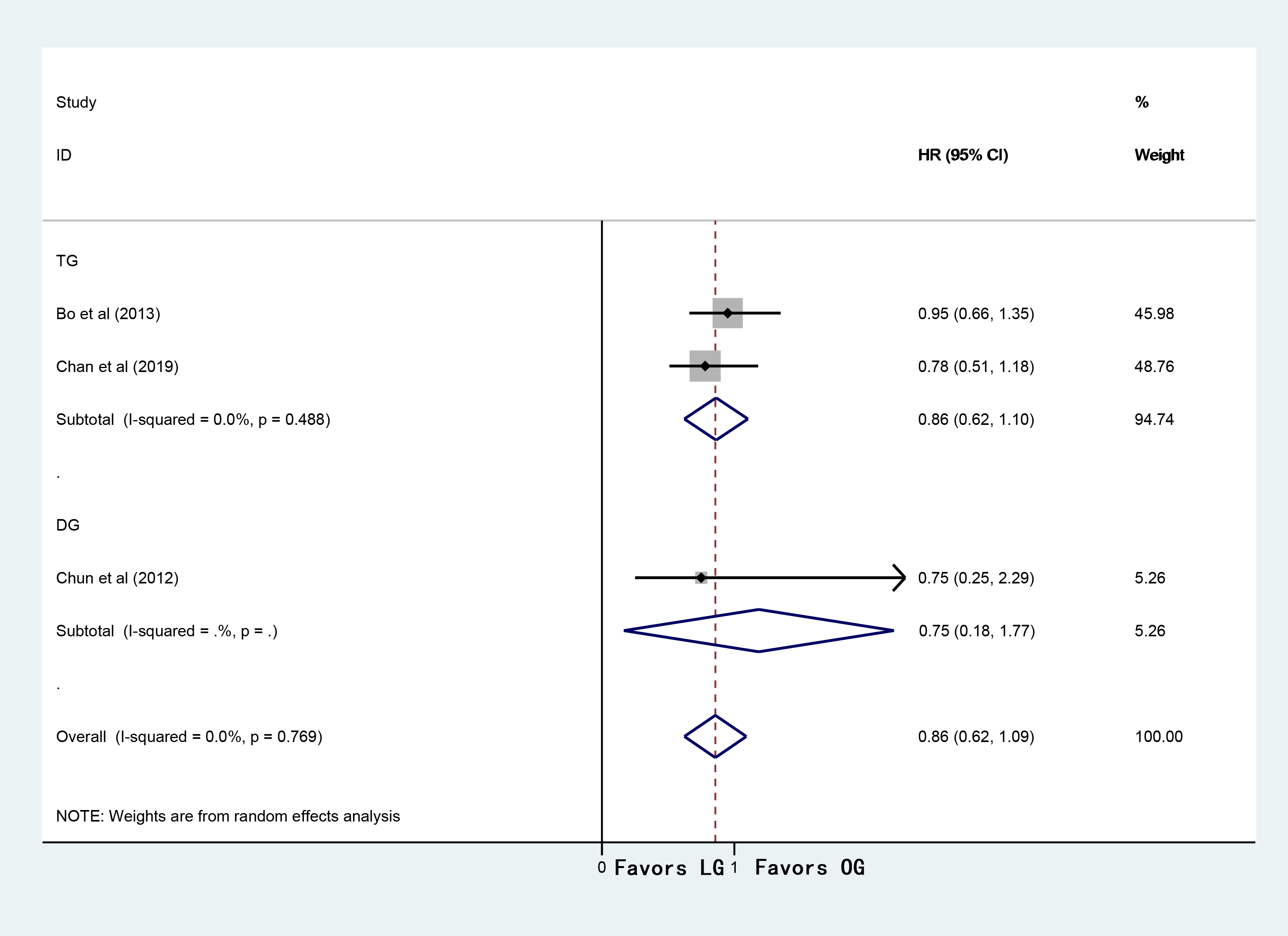

Supplement: Supplementary file 7 — Additional file 7: Figure S7. Subgroup analysis based on operative procedure for 5-year overall survival. [file 12957_2020_1888_MOESM7_ESM.tif]

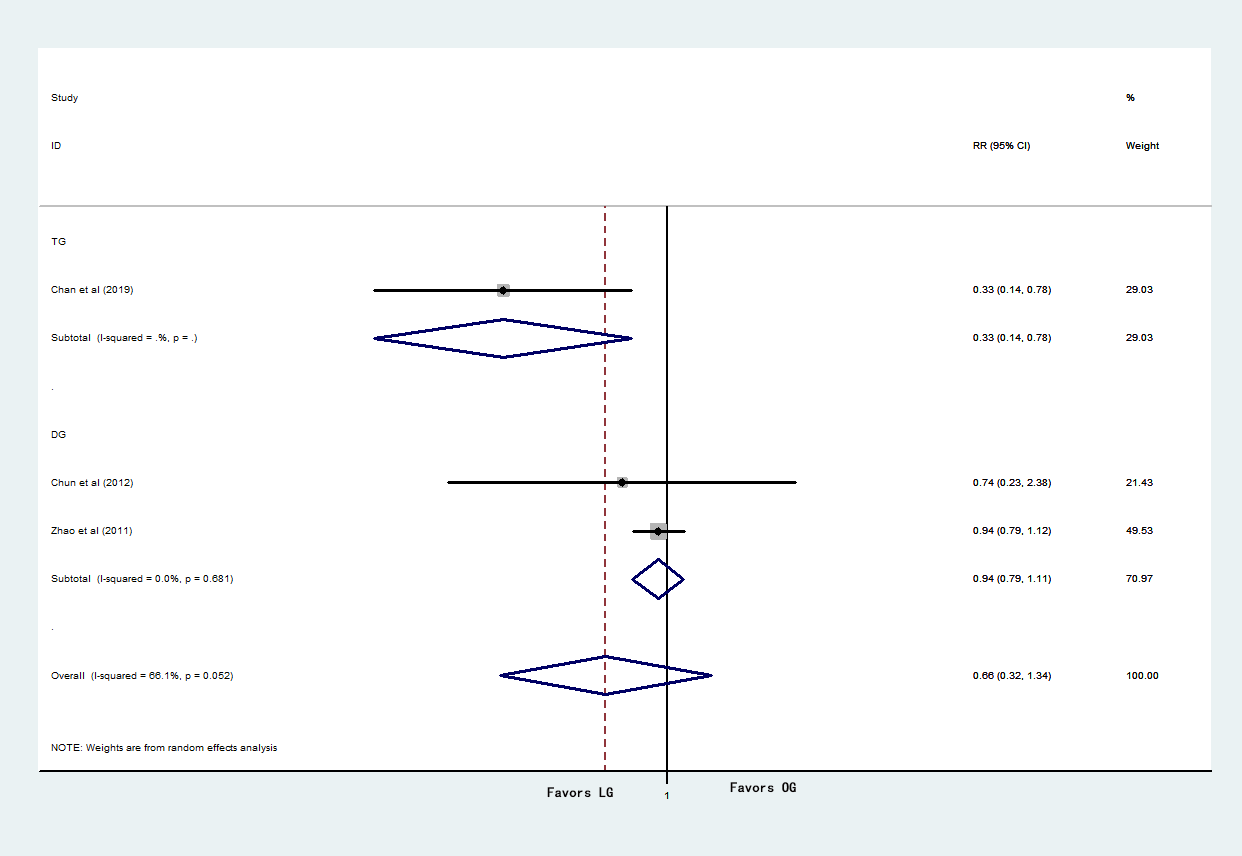

Supplement: Supplementary file 8 — Additional file 8: Figure S8. Subgroup analysis based on operative procedure for tumor recurrence. [file 12957_2020_1888_MOESM8_ESM.tif]
